# Proteomic analysis of human spermatozoa proteins with oxidative stress

**DOI:** 10.1186/1477-7827-11-48

**Published:** 2013-05-20

**Authors:** Rakesh Sharma, Ashok Agarwal, Gayatri Mohanty, Alaa J Hamada, Banu Gopalan, Belinda Willard, Satya Yadav, Stefan du Plessis

**Affiliations:** 1 Center for Reproductive Medicine, Glickman Urological and Kidney Institute, Cleveland Clinic, Cleveland, OH, USA; 2 Bioinformatics Core Services, Lerner Research Institute, Cleveland Clinic, Cleveland, OH, USA; 3 Proteomic Core Lab, Lerner Research Institute, Cleveland Clinic, Cleveland, OH, USA; 4 Molecular Biotechnology Core lab, Lerner Research Institute, Cleveland Clinic, Cleveland, OH, USA; 5 Medical Physiology, Stellenbosch University, Tygerberg, South Africa; 6 Permanent address: Ravenshaw University, Cuttack, Odisha, India

**Keywords:** Spermatozoa, Reactive oxygen species, Oxidative stress, Proteomics, Male infertility

## Abstract

**Background:**

Oxidative stress plays a key role in the etiology of male infertility. Significant alterations in the sperm proteome are associated with poor semen quality. The aim of the present study was to examine if elevated levels of reactive oxygen species cause an alteration in the proteomic profile of spermatozoa.

**Methods:**

This prospective study consisted of 52 subjects: 32 infertile men and 20 normal donors. Seminal ejaculates were classified as ROS+ or ROS- and evaluated for their proteomic profile. Samples were pooled and subjected to LC-MS/MS analysis through in-solution digestion of proteins for peptide characterization. The expression profile of proteins present in human spermatozoa was examined using proteomic and bioinformatic analysis to elucidate the regulatory pathways of oxidative stress.

**Results:**

Of the 74 proteins identified, 10 proteins with a 2-fold difference were overexpressed and 5 were underexpressed in the ROS+ group; energy metabolism and regulation, carbohydrate metabolic processes such as gluconeogenesis and glycolysis, protein modifications and oxidative stress regulation were some of the metabolic processes affected in ROS+ group.

**Conclusions:**

We have identified proteins involved in a variety of functions associated with response and management of oxidative stress. In the present study we focused on proteins that showed a high degree of differential expression and thus, have a greater impact on the fertilizing potential of the spermatozoa. While proteomic analyses identified the potential biomarkers, further studies through Western Blot are necessary to validate the biomarker status of the proteins in pathological conditions.

## Background

Male-factor infertility contributes to 50% of infertile couples worldwide, and while the causes are multifactorial, current research has focused on oxidative stress. Specifically, oxidative stress may lead to alterations in protein expression levels in spermatozoa, causing molecular and genetic defects. Research has already shown that oxidative stress is associated with a variety of male infertility diagnosis such as varicocele, idiopathic infertility, spinal cord injury, prostatitis and leukocytospermia [1-8]. Varicocele is implicated in 35% of men with primary infertility and in 80% of infertile men with secondary infertility, and oxidative stress is one of the many causes of varicocele related infertility [9-11]. Oxidative stress in men with varicocele is also associated both with motility and grade of varicocele.

Understanding the protein profile of spermatozoa is essential for identifying the protein alterations that occur as a result of increased production of ROS and for better diagnosis of male infertility. The development of 2-dimensional-polyacrylamide gel electrophoresis (2-DE) coupled with Western blot have aided in the identification of proteins, in relation to sperm composition and function [12,13]. Furthermore advancements in mass spectrometry analyses of spermatozoa have broadened our knowledge and aided in the identification of pivotal sperm proteins [14,15]. Numerous studies have reported the presence of proteins in studies involving human spermatozoa through the implementation of proteomic techniques such as 2-DE, liquid chromatography tandem mass spectrometry (LC-MS/MS), matrix assisted laser desorption and ionization- time of flight tandem mass spectrometry (MALDI-TOF-MS/MS), and 2-dimensional difference in gel electrophoresis (DIGE) [16-21].

In this comparative study, we employed LC- MS/ MS and functional bioinformatic analyses to identify the relative abundance of proteins in spermatozoa obtained from seminal ejaculates of infertile men who were referred to the andrology clinical laboratory for advanced semen analysis (including parameters of oxidative stress such as levels of ROS in spermatozoa, concentration of antioxidant in the seminal plasma and the extent of DNA damage). We also included healthy men with high and low levels of ROS in their seminal ejaculates compared to those with low levels of ROS. Oxidative stress plays a major role in male infertility, but the crucial proteins that are differentially expressed in ROS are not known. Understanding the role of these proteins is therefore important in our understanding of the post-genomic events. It will help identify potential proteins that could serve as biomarkers to better manage and treat oxidative stress, and consequently, male infertility.

## Methods

The study was approved by the Institutional Review Board of Cleveland Clinic and was conducted in accordance with national and international guidelines. A total of 52 subjects were enrolled in the study. Twenty healthy male volunteers of unproven fertility (normal semen analysis results but had not established a pregnancy) were selected on the basis of normal semen analysis. Thirty two infertile men attending our infertility clinic were referred to the andrology lab for advanced semen parameters of oxidative stress. The breakdown of the infertile subjects was as follows: primary infertility: n = 25 (25/ 32; 78.1%); Secondary infertility: n = 7 (7/25; 28%). Of the 25 men with primary infertility, 64% (16/ 25) had <2 years and 36% (9/ 25) >2 years. Clinical varicocele (grade 1-2) was present in 10 (62.5%) of the 16 men who presented with <2 years of primary infertility and in 5 (55.6%) of the 9 men with >2 years of primary infertility. In the 7 patients with secondary infertility, 6 (85.7%) had a clinical varicocele (grade 1-2).

Semen samples were collected by masturbation after 2-3 days of sexual abstinence and analyzed according to WHO 2010 criteria [22]. A complete semen analysis was performed on both donors and patients and included both macroscopic (volume, pH, color, viscosity, liquefaction time, split or complete ejaculate) and microscopic parameters (concentration, motility, morphology, round cells and peroxidase or Endtz test). All subjects were also examined for levels of reactive oxygen species (ROS) in the seminal ejaculate. The remainder of the seminal ejaculate was frozen without any cryoprotectant and stored at -55°C for proteomic analysis.

### Semen analysis

After liquefaction, complete semen analysis was performed to evaluate the sperm parameters (sperm count, percentages motility and morphology), according to WHO guidelines [22]. Semen analysis was done using a MicroCell counting chamber (Vitrolife, San Diego, CA). Smears of the raw semen were stained with a Diff-Quik kit (Baxter Healthcare Corporation, Inc., McGaw Park, IL) for assessment of sperm morphology according to WHO criteria.

### Measurement of reactive oxygen species

In the freshly ejaculated and completely liquefied semen, ROS levels were measured by a chemiluminescence assay using luminol (5-amino-2, 3- dihydro-1, 4-phthalazinedione). Test samples consisted of luminol (10 μL, 5 mM) and 400 μL of semen. Negative controls were prepared by replacing the sperm suspension with phosphate buffered saline. Chemiluminescence was measured for 15 min using a Berthold luminometer (Autolumat Plus 953). Results were expressed as relative light units (RLU)/sec/ × 10^6^ sperm. Reference value for ROS – group was: <20 RLU/s/×10^6^ sperm. ROS levels >20 RLU/s/×10^6^ sperm were considered positive [23].

### Separation of spermatozoa and In-solution Digestion of Proteins

We were interested in examining the effect of ROS levels on the distribution / alteration of proteins in an effort to understand the underlying mechanism of infertility. Based on our published reference levels, all samples with ROS levels below 20 RLU/sec/ × 10^6^ sperm were considered to be ROS – and those with ROS levels >20 RLU/sec/ × 10^6^ sperm were considered to be ROS + [23]. For proteomic analysis, frozen seminal ejaculates from both the donors and patients were divided into 2 groups: ROS+ and ROS-, irrespective of the subject’s clinical diagnoses. Samples were centrifuge at 10,000 g for 10 minutes to pellet the spermatozoa and the clear seminal plasma was discarded. Samples were pooled into ROS+ and ROS – groups and solubilized in lysis buffer containing 2% octyl-β-glucopyranoside, 100 mM dithiothreitol (DTT), 9.8 M urea and protease inhibitors [12]. Spermatozoa samples were stored overnight in the refrigerator to allow for complete lysis of the spermatozoa. The samples were first precipitated in cold acetone, solubilized in 6 M urea, reduced with DTT, and alkylated with iodoacetamide. The samples were subsequently diluted to give a urea concentration <2 M and then digested with trypsin. The tryptic digestion was subjected to a C18 clean up and then brought up in 50 μL of 1% acetic acid [12].

### Liquid chromatography- Mass Spectrometer analysis (LC-MS-MS)

The LC-MS/MS system was a Finnigan LTQ linear ion trap mass spectrometer system (Thermo Finnigan). Ten μL volumes of the extract were injected on a self-packed high performance liquid chromatography column (Phenomenex Jupiter C18 reversed-phase capillary chromatography column). Peptides were eluted from the column by an acetonitrile/0.1% formic acid gradient at a flow rate of 0.25 μL/min. They were introduced into the source of the mass spectrometer on-line. The microelectrospray ion source was operated at 2.5 kV. The digest was analyzed using the data dependent multitask capability of the instrument acquiring full scan mass spectra to determine peptide molecular weights and product ion spectra to determine amino acid sequence in successive instrument scans [12].

### Data analysis

The data were analyzed using all collision- induced dissociation spectra collected in the experiment to search the National Center for Biotechnology Information (NCBI) human reference sequence database with the search program MASCOT (Matrix Science, Boston, MA). Mascot is a mass spectral search algorithm that uses mass spectrometry data to identify proteins from primary sequence databases. These searches were used to identify the proteins present in the in-solution digestions. After identification, a database consisting of all proteins identified in these searches was created and used for a second set of searches performed with SEQUEST (Thermo Finnigan). SEQUEST is a tandem mass spectrometry data analysis program used for protein identification. Sequest identifies collections of tandem mass spectra to peptide sequences that have been generated from databases of protein sequences. The results from these SEQUEST searches were used to determine the spectral counts. These spectral count values were normalized by the total number of spectral counts for all proteins in the sample and the number of amino acids present in the protein. As the precision of the proteomic analysis has an average error of 10-20%, a 2-fold change in protein expression was considered significant.

Furthermore functional bioinformatics analysis were done using publicly available (Gene Ontology (GO) annotations from GO Term Finder [24] and GO Term Mapper [25], UNIPROT [26], STRAP [27] and BioGPS [28] and proprietary software packages (Ingenuity Pathway Analysis (IPA) from Ingenuity® Systems [29] and Metacore™ from GeneGo Inc. [30] to identify the differentially affected processes, pathways, interactions and cellular distribution of the proteins in the two study groups.

## Results

### Semen parameters and oxidative stress parameters

Most of the infertile men were diagnosed with primary infertility and varicocele. The median and 25^th^ and 75^th^ percentile for sperm concentration (×10^6^/ mL) in controls was 64.9 (45.4-74.9) versus patients 23.0 (13.0-41.4). The median and 25^th^ and 75^th^ percentile motility in controls was 58.3(50.0-65.7) compared to infertile men 50.5 (36.8-58.0). Among the patients, 46.8% (15/32) had > 1 × 10^6^ round cells / mL compared to donors 35% (7/20) of the donors, of these 15 patients, 15.6% were positive for the Endtz test – a marker for granulocytes, which are the major contributors of ROS production. None of the donors was positive for the Endtz test. An overlap in morphology was seen in both the donors (3.5% ± 1.6%) and infertile men (3.6% ± 3.2%).

Among the patients 22 of 32 (68.8%) were positive for ROS compared to 9 of 20 (45%) in the controls. Levels of ROS were significantly lower (RLU/s/×10^6^ sperm) median and 25^th^ and 75^th^ percentile were seen in donors [4.4(0, 300.2)] compared to infertile men [104(0, 1341)]. Among the primary infertility patients with <2 years of infertility, 80% were positive for ROS and 55.6% were positive in the patients with >2 years of primary infertility. A significantly larger percentage of men (83%) were positive for ROS in those with secondary infertility. Teratozoospermia was present in 48% of men with primary infertility.

When all subjects were categorized into ROS+ and ROS- groups, ROS levels (RLU/s/10^6^sperm: median, 25^th^, 75^th^ percentile) were significantly different and higher in the ROS+ group [2236(33, 4439)] group compared to the ROS – group [4 (0, 9); P < 0.01)].

### MS identification of the differentially expressed proteins in ROS+ and ROS- samples

In this study, we analyzed the differential expression of proteins in spermatozoa obtained from seminal ejaculates with high levels of ROS+ and those with normal levels (ROS-). Tables 1 and 2 show the primary proteins with their NCBI database index number, calculated molecular weight, isoelectric point (pI), peptide coverage and Mascot scores for ROS+ and ROS- groups. Based on the SEQUEST score, a total of 74 proteins were identified and differential expression was calculated based on the normalized spectral count ratios between ROS+ and ROS-. In the ROS+ group, proteins that had >50% peptide coverage were: A-Kinase anchor protein 4 isoform 2; Lactotransferrin precursor; Tubulin, beta, 2; Tubulin, beta 4; Tubulin, alpha 3c; Ropporin, rhophilin associated protein 1B; Prolactin-induced protein; Histone cluster 1, H2aa; Beta actin and Triosephosphate isomerase 1 isoform 1. In the ROS- group, proteins that had >50% peptide coverage were: A-Kinase anchor protein 4 isoform 2; Lactotransferrin precursor; Spermatogenic Tubulin, beta, 2 precursor; Tubulin, beta 4; Tubulin, alpha 3c; Ropporin, rhophilin associated protein 1B; Prolactin-induced protein; Histone cluster 1, H2aa and Triosephosphate isomerase 1 isoform 1;

**Table 1:** **Protein name**, **NCBI database index number**, **calculated MW**, **pI**, **peptide coverage and Mascot score in ROS**+ **group**

**No.**	**Protein name**	**NCBI database index number**	**Calculated MW (kDa)**	**pI**	**LTQ**	
					**Peptides**	**Mascot**
					**(Coverage)**	**score**
1	A-Kinase anchor protein 4 isoform 2	21493039	94	6.7	34 (54%)	10988
2	lactotransferrin precursor	54607120	80	8.5	36 (64%)	7287
3	tubulin, beta, 2	5174735	50	4.7	24 (68%)	6702
4	tubulin, beta 4	21361322	50	4.8	19 (57%)	5071
5	glyceraldehyde-3-phosphate dehydrogenase, spermatogenic	7657116	44	8.4	12 (48%)	5711
6	tubulin, alpha 3c	17921993	50	4.9	15 (51%)	2942
7	ropporin, rhophilin associated protein 1B	59891409	24	5.1	7 (56%)	2587
8	semenogelin II precursor	4506885	65	9.1	13 (22%)	2277
9	outer dense fiber of sperm tails 2 isoform 2	24430183	73	7.2	16 (31%)	2051
10	semenogelin I isoform a preproprotein	4506883	52	9.3	12 (31%)	1983
11	sperm protein associated with the nucleus, X chromosome, family member C	13435137	11	5.0	3 (45%)	1892
12	tubulin, alpha, ubiquitous	57013276	50	4.9	13 (46%)	1616
13	glutathione S-transferase mu 3	23065552	26	5.3	7 (37%)	1595
14	prolactin-induced protein	4505821	16	8.2	8 (62%)	1559
15	A Kinase anchor protein 3	21493041	95	5.8	20 (35%)	1416
16	histone cluster 1, H2ba	24586679	14	10.3	1 (11%)	1371
17	mitochondrial malate dehydrogenase precursor	21735621	35	8.9	8 (33%)	1342
18	histone cluster 1, H2aa	25092737	14	10.8	6 (58%)	1334
19	clusterin isoform 1	42716297	58	6.2	6 (16%)	1204
20	ropporin	21359920	24	5.5	6 (43%)	1184
21	sorbitol dehydrogenase	156627571	38	8.2	7 (35%)	1095
22	mitochondrial ATP synthase beta subunit precursor	32189394	56	5.2	16 (53%)	976
23	beta actin	4501885	42	5.2	9 (54%)	909
24	triosephosphate isomerase 1 isoform 1	4507645	26	6.4	9 (55%)	761
25	glyceraldehyde-3-phosphate dehydrogenase	7669492	36	8.5	6 (32%)	706
26	L-lactate dehydrogenase C– 1 peptide	4504973	36	7.0	1 (5%)	674
27	fatty acid synthase	41872631	275	6.0	3 (8%)	669
28	sperm autoantigenic protein 17	8394343	17	4.7	2 (22%)	592
29	pyruvate kinase, muscle isoform M2	33286418	58	7.9	11 (38%)	560
30	ATP synthase, H + transporting, mitochondrial F1 complex, alpha subunit precursor	4757810	59	9.1	8 (23%)	542
31	brain creatine kinase	21536286	42	5.3	7 (32%)	511
32	heat shock protein 90 kDa beta, member 1	4507677	92	4.7	6 (10%)	502
33	heat shock 70 kDa protein 2	13676857	70	5.5	10 (21%)	448
34	heat shock 90 kDa protein 1, alpha isoform 1	153792590	98	5.0	8 (13%)	447
35	saccharopine dehydrogenase (putative)	55770836	47	9.2	5 (29%)	395
36	actin, alpha 1, skeletal muscle	4501881	42	5.2	6 (36%)	374
37	glutamine synthetase	19923206	42	6.4	3 (9%)	374
38	fructose-bisphosphate aldolase A	4557305	39	8.3	5 (29%)	366
39	eukaryotic translation elongation factor 1 alpha 1	4503471	50	9.1	8 (36%)	347
40	fibronectin 1 isoform 3 preproprotein	16933542	262	5.4	6 (4%)	321
41	prostate, ovary, testis expressed protein on chromosome 2	153791352	123	5.8	4 (8%)	317
42	acetyl-Coenzyme A acetyltransferase 1 precursor	4557237	45	8.9	9 (33%)	316
43	voltage-dependent anion channel 2	42476281	32	7.4	4 (22%)	293
44	outer dense fiber of sperm tails 1	194248060	30	8.4	4 (26%)	283
45	phosphoglycerate kinase 2	31543397	45	8.7	5 (30%)	253
46	histone cluster 1, H2ae	10645195	14	11.0	4 (49%)	251
47	protein disulfide – isomerase A3 precursor	21361657	57	5.9	4 (13%)	243
48	acid phosphatase, prostate short isoform precursor	6382064	44	5.8	5 (16%)	240
49	voltage-dependent anion channel 3 isoform b	25188179	30	8.8	5 (27%)	226
50	phospholipase A2, group IIA precursor	4505849	16	9.4	2 (23%)	202
51	transglutaminase 4 (prostate)	156627577	77	6.3	8 (17%)	187
52	glutathione peroxidase 4 isoform A precursor	75709200	22	8.6	4 (32%)	181
53	heat shock 70 kDa protein 5	16507237	72	5.0	4 (8%)	161
54	heat shock protein beta-1	4504517	22	5.9	3 (31%)	160
55	angiotensin I converting enzyme 1 isoform 1 precursor	4503273	150	5.9	3 (4%)	160
56	peroxiredoxin 6	4758638	25	6.0	2 (16%)	145
57	chaperonin containing TCP1, subunit 5 (epsilon)	24307939	60	5.4	4 (12%)	142
58	valosin-containing protein	6005942	89	5.1	3 (7%)	142
59	sperm acrosomal membrane protein 14 – 1 peptide	19424138	13	5.4	1 (11%)	132
60	chaperonin containing TCP1, subunit 8 (theta)	48762932	60	5.4	4 (14%)	131
61	L-lactate dehydrogenase A isoform 1	5031857	36	8.4	2 (13%)	124
62	phosphoglycerate dehydrogenase	23308577	57	6.2	3 (13%)	112
63	clathrin heavy chain 1	4758012	193	5.4	4 (5%)	108
64	chaperonin containing TCP1, subunit 4 (delta)	38455427	58	7.9	5 (21%)	99
65	peptidylprolyl isomerase A	10863927	18	7.6	2 (23%)	96
66	fumarate hydratase precursor	19743875	54	8.8	8 (31%)	92
67	RAB2A, member RAS oncogene family	4506365	23	6.0	3 (18%)	92
68	olfactomedin 4 precursor	32313593	57	5.5	2 (5%)	90

**Table 2:** **Protein name**, **NCBI database index number**, **calculated MW**, **pI**, **peptide coverage and Mascot score in ROS**- **group**

**No**.	**Protein name**	**NCBI database index number**	**Calculated MW (kDa)**	**pI**	**LTQ**	
					**Peptides (Coverage)**	**Mascot score**
1	A-Kinase anchor protein 4 isoform 2	21493039	94	6.7	34 (54%)	11284
2	lactotransferrin precursor	54607120	80	8.5	32 (56%)	6049
3	gyceraldehyde-3-phosphate dehydrogenase,	7657116	44	8.4	12 (48%)	5959
4	spermatogenic tubulin, beta, 2 precursor	5174735	50	4.7	25 (68%)	5842
5	semenogelin I isoform a preproprotein	4506883	52	9.3	11(24%)	4965
6	tubulin, beta 4	21361322	50	4.8	19 (58%)	4879
7	semenogelin II precursor	4506885	65	9.1	15 (25%)	4205
8	tubulin, alpha 3c	17921993	50	4.9	15 (56%)	2645
9	ropporin, rhophilin associated protein 1B	59891409	24	5.1	6 (53%)	2427
10	prolactin-induced protein	4505821	16	8.2	8 (62%)	2164
11	A-kinase anchor protein 3	21493041	95	5.8	16 (28%)	1800
12	outer dense fiber of sperm tails 2 Isoform 2	24430183	73	7.2	16 (31%)	1722
13	sperm protein associated with the Nucleus, X chromosome, family Member C	13435137	11	5.0	3 (45%)	1437
14	tubulin alpha 6	14389309	50	4.9	11 (45%)	1374
15	ropporin	21359920	24	5.5	6 (43%)	1280
16	clusterin isoform 1	42716297	58	6.2	5 (15%)	1257
17	sorbitol dehydrogenase	156627571	38	8.2	4 (23%)	1249
18	beta actin	4501885	42	5.2	6 (34%)	1232
19	pyruvate kinase, muscle isoform M1	3328642	58	7.6	13 (47%)	1133
20	histone cluster 1, H2aa	25092737	14	10.8	4 (51%)	1121
21	mitochondrial ATP synthase beta subunit precursor	32189394	56	5.2	13 (38%)	1031
22	fatty acid synthase	41872631	275	6.0	10 (9%)	935
23	glutathione S-transferase mu 3	23065552	26	5.3	7 (32%)	929
24	histone cluster 1, H2ba -1 peptide	24586679	14	10.3	1 (11%)	823
25	fibronectin 1 isoform 3 preproprotein	16933542	262	5.4	16 (11%)	790
26	L-lactate dehydrogenase C	4504973	36	7.0	2 (7%)	693
27	heat shock 90 kDa protein 1, alpha isoform 2	154146191	85	4.9	10 (18%)	682
28	glyceraldehyde-3-phosphate dehydrogenase	7669492	36	8.5	3 (15%)	621
29	sperm autoantigenic protein 17	8394343	17	4.7	2 (22%)	552
30	triosephosphate isomerase 1 isoform 1	4507645	26	6.4	6 (61%)	530
31	mitochondrial malate dehydrogenase precursor	21735621	35	8.9	5 (23%)	512
32	phosphoglycerate kinase 2	31543397	45	8.7	4 (17%)	506
33	heat shock 70 kDa protein 2	13676857	70	5.5	8 (22%)	423
34	histone cluster 1, H2ae	10645195	14	11.0	3 (43%)	392
35	acetyl-Coenzyme A acetyltransferase 1 precursor	4557237	45	8.9	8 (34%)	388
36	eukaryotic translation elongation factor 1 alpha 1	4503471	50	9.1	7 (30%)	383
37	ATP synthase, H + transporting, mitochondrial F1 complex, alpha subunit precursor	4757810	59	9.1	8 (22%)	378
38	outer dense fiber of sperm tails 1	194248060	30	8.4	3 (22%)	334
39	voltage-dependent anion channel 2	42476281	32	7.4	3 (19%)	332
40	acid phosphatase, prostate short isoform precursor	6382064	44	5.8	5 (16%)	326
41	brain creatine kinase	21536286	42	5.3	7 (29%)	268
42	saccharopine dehydrogenase (putative)	55770836	47	9.2	4 (29%)	265
43	protein disulfide-isomerase A3 precursor	21361657	57	5.9	2 (9%)	261
44	eukaryotic translation elongation factor 2	4503483	96	6.4	3 (8%)	261
45	heat shock protein 90 kDa beta, member 1	4507677	92	4.7	5 (8%)	250
46	heat shock protein beta-1	4504517	22	5.9	4 (36%)	191
47	glutamine synthetase	19923206	42	6.4	3 (14%)	179
48	valosin-containing protein	6005942	89	5.1	7 (18%)	177
49	phospholipase A2, group IIA precursor	4505849	16	9.4	2 (23%)	170
50	tyrosine 3/tryptophan 5 -monooxygenase activation protein, zeta polypeptide	4507953	27	4.7	2 (19%)	167
51	L-lactate dehydrogenase A isoform 1	5031857	36	8.4	2 (7%)	167
52	fructose-bisphosphate aldolase A	4557305	39	8.3	3 (16%)	167
53	chaperonin containing TCP1, subunit 5 (epsilon)	24307939	60	5.4	3 (9%)	157
54	voltage-dependent anion channel 3 isoform b	25188179	30	8.8	4 (26%)	147
55	peroxiredoxin 6	4758638	25	6.0	4 (33%)	140
56	glutathione peroxidase 4 isoform A precursor	75709200	22	8.6	5 (43%)	139
57	transglutaminase 4 (prostate)	156627577	77	6.3	5 (9%)	136
58	enolase 1	4503571	47	7.0	3 (12%)	134
59	T-complex protein 1 isoform b	57863259	44	7.5	5 (30%)	129
60	CSE1 chromosome segregation 1-like protein	29029559	111	5.5	2 (3%)	128
61	fumarate hydratase precursor	19743875	54	8.8	3 (12%)	115
62	lectin, mannose-binding 2 precursor	5803023	40	6.4	2 (13%)	115
63	clathrin heavy chain 1	4758012	193	5.4	6 (7%)	111
64	chaperonin containing TCP1, subunit 4 (delta)	38455427	58	7.9	4 (19%)	107
65	RAB2A, member RAS oncogene family	4506365	23	6.0	3 (18%)	106
66	chromosome 20 open reading frame 3	24308201	46	5.8	3 (19%)	105

Out of the total 74 proteins identified, 47 were overexpressed and 27 underexpressed in the ROS+ group; 15 proteins were significantly (2-fold) differentially expressed in the ROS+ samples compared to the ROS- samples.

Of the differentially expressed proteins, 15 proteins were overexpressed in the ROS+ samples while 5 were underexpressed in comparison to ROS- samples (Table 3). The overexpressed proteins included the histone cluster1; H2ba (HIST1H2BA); mitochondrial malate dehydrogenase precursor (MDH2); heat shock protein 90 kDa beta, member 1 (HSP90B1); heat shock 70 kDa protein 5 (HSPA5); glutamine synthetase (GLUL); transglutaminase 4 (prostate) (TGM4); glutathione peroxidase 4 isoform A precursor (GPX4); sperm acrosomal membrane protein 14 (SPACA4); olfactomedin 4 precursor (OLFM4) and chromosome 20 open reading frame 3 (C20orf3). Proteins that were found to be under expressed were: semenogelin II precursor (SEMG2); peroxiredoxin 6 (PRDX6); clathrin heavy chain 1 (CLTC); eukaryotic translation elongation factor 2 (EEF2) and enolase 1 (ENO1).

**Table 3:** **Differentially expressed proteins in ROS**- **and ROS**+ **groups along with the spectral count and the spectral count ratio for the two groups**

**Protein**	**NCBI #**	**SC ROS-**	**NSC-ROS-**	**SC ROS+**	**NSC-ROS+**	**NSC ratio in ROS+/ROS-**
acetyl-Coenzyme A acetyltransferase 1 precursor (ACAT1)	4557237	20	0.006	26.000	0.008	1.4
acid phosphatase, prostate short isoform precursor (ACPP)	6382064	17	0.005	27.000	0.009	1.7
actin, alpha 1, skeletal muscle (ACTA1)	4501881	0	0.000	8.000	0.003	**ROS** + **only**
A-kinase anchor protein 3 (AKAP3)	21493041	76	0.022	70.000	0.022	1.0
A-kinase anchor protein 4 isoform 2 (AKAP4)	21493039	497	0.145	467.000	0.147	1.0
angiotensin I converting enzyme 1 isoform 1 precursor (ACE)	4503273	5	0.001	5.000	0.002	1.1
ATP synthase, H + transporting, mitochondrial F1 complex, alpha subunit precursor (ATP5A1)	4757810	28	0.008	30.000	0.009	1.2
beta actin (ACTB)	4501885	53	0.015	36.000	0.011	0.7
brain creatine kinase (CKB)	21536286	19	0.006	21.000	0.007	1.2
chaperonin containing TCP1, subunit 4 (delta) (CCT4)	38455427	4	0.001	6.000	0.002	1.6
chaperonin containing TCP1, subunit 5 (epsilon) (CCT8)	24307939	4	0.001	7.000	0.002	1.9
chaperonin containing TCP1, subunit 8 (theta)	48762932	5	0.001	4.000	0.001	0.9
chromosome 20 open reading frame 3 (C20orf3)	24308201	2	0.001	4.000	0.001	**2**.**2**
clathrin heavy chain 1 (CLTC)	4758012	14	0.004	6.000	0.002	**0**.**5**
clusterin preproprotein (CLU)	355594753	56	0.016	55.000	0.017	1.1
CSE1 chromosome segregation 1-like protein (CSE1L)	29029559	3	0.001	0.000	0.000	**ROS**- **only**
enolase 1 (ENO1)	4503571	7	0.002	2.000	0.001	**0**.**3**
eukaryotic translation elongation factor 1 alpha 1 (EEF1A1)	4503471	28	0.008	33.000	0.010	1.3
eukaryotic translation elongation factor 2 (EEF2)	4503483	12	0.003	5.000	0.002	**0**.**5**
fatty acid synthase (FASN)	41872631	40	0.012	35.000	0.011	1.0
fibronectin 1 isoform 3 preproprotein (FN1)	16933542	41	0.012	21.000	0.007	0.6
fructose-bisphosphate aldolase A (ALDOA)	4557305	15	0.004	17.000	0.005	1.2
fumarate hydratase precursor (FH)	19743875	5	0.001	7.000	0.002	1.5
glutamine synthetase (GLUL)	19923206	4	0.001	12.000	0.004	**3**.**2**
glutathione peroxidase 4 isoform A precursor (GPX4)	75709200	8	0.002	21.000	0.007	**2**.**8**
glutathione S-transferase mu 3 (GSTM3)	23065552	50	0.015	65.000	0.020	1.4
glyceraldehyde-3-phosphate dehydrogenase (GAPDH)	7669492	14	0.004	22.000	0.007	1.7
glyceraldehyde-3-phosphate dehydrogenase, spermatogenic (GAPDHS)	7657116	210	0.061	185.000	0.058	1.0
heat shock 70 kDa protein 2 (HSPA2)	13676857	26	0.008	22.000	0.007	0.9
heat shock 70 kDa protein 5 (HSPA5)	16507237	5	0.001	11.000	0.003	**2**.**4**
heat shock 90 kDa protein 1, alpha isoform 1 (HSP90AA1)	153792590	39	0.011	26.000	0.008	0.7
heat shock protein 90 kDa beta, member 1 (HSP90B1)	4507677	11	0.003	22.000	0.007	**2**.**2**
heat shock protein beta-1 (HSPB1)	4504517	5	0.001	4.000	0.001	0.9
histone cluster 1, H2aa (HIST1H2AA)	25092737	47	0.014	56.000	0.018	1.3
histone cluster 1, H2ae (HISTH1AE)	10645195	14	0.004	13.000	0.004	1.0
histone cluster 1, H2ba (HIST1H2BA)	24586679	22	0.006	49.000	0.015	**2**.**4**
lactotransferrin precursor (LTF)	54607120	246	0.072	292.000	0.092	1.3
lectin, mannose-binding 2 precursor	5803023	3	0.001	5.000	0.002	1.8
L-lactate dehydrogenase A isoform 1 (LDHA)	5031857	10	0.003	9.000	0.003	1.0
L-lactate dehydrogenase C (LDHC)	4504973	17	0.005	12.000	0.004	0.8
mitochondrial ATP synthase beta subunit precursor (ATP5B)	32189394	50	0.015	70.000	0.022	1.5
mitochondrial malate dehydrogenase precursor (MDH2)	21735621	22	0.006	44.000	0.014	**2**.**2**
olfactomedin 4 precursor (OLFM4)	32313593	1	0.000	4.000	0.001	**4**.**3**
outer dense fiber of sperm tails 1 (ODF1)	194248060	15	0.004	14.000	0.004	1.0
outer dense fiber of sperm tails 2 isoform 2 (ODF2)	24430183	90	0.026	91.000	0.029	1.1
peptidylprolyl isomerase A (PPIA)	10863927	4	0.001	2.000	0.001	0.5
peroxiredoxin 6 (PRDX6)	4758638	4	0.001	1.000	0.0003	**0**.**3**
phosphoglycerate dehydrogenase (PHGDH)	23308577	3	0.001	5.000	0.002	1.8
phosphoglycerate kinase 2 (PGK2)	31543397	17	0.005	9.000	0.003	0.6
phospholipase A2, group IIA precursor (PLA2G2A)	4505849	6	0.002	10.000	0.003	1.8
prolactin-induced protein (PIP)	4505821	118	0.034	101.000	0.032	0.9
prostate specific antigen isoform 1 preproprotein (KLK3)	4502173	3	0.001	2.000	0.001	0.7
protein disulfide-isomerase A3 precursor (PDIA3)	21361657	7	0.002	7.000	0.002	1.1
pyruvate kinase, muscle isoform M2 (PKM)	33286418	49	0.014	31.000	0.010	0.7
RAB2A, member RAS oncogene family (RAB2A)	4506365	3	0.001	3.000	0.001	1.1
ropporin (ROPN1)	21359920	20	0.006	31.000	0.010	1.7
ropporin, rhophilin associated protein 1B (ROPN1B)	59891409	73	0.021	73.000	0.023	1.1
saccharopine dehydrogenase (putative) (SCCPDH)	55770836	13	0.004	16.000	0.005	1.3
semenogelin I isoform a preproprotein (SEMG1)	4506883	171	0.050	101.000	0.032	0.6
semenogelin II precursor (SEMG2)	4506885	513	0.149	226.000	0.071	**0**.**5**
sorbitol dehydrogenase (SORD)	156627571	36	0.010	27.000	0.009	0.8
sperm acrosomal membrane protein 14	19424138	1	0.000	2.000	0.001	**2**.**2**
sperm autoantigenic protein 17 (SPA17)	8394343	15	0.004	20.000	0.006	1.4
sperm protein associated with the nucleus, X chromosome, family member C (SPANX)	13435137	54	0.016	80.000	0.025	1.6
transglutaminase 4 (prostate) (TGM4)	156627577	4	0.001	9.000	0.003	**2**.**4**
triosephosphate isomerase 1 isoform 1 (TPI1)	4507645	28	0.008	31.000	0.010	1.2
tubulin alpha 6 (TUBA1C)	14389309	9	0.003	11.000	0.003	1.3
tubulin, alpha 3c (TUBA3C)	17921993	117	0.034	134.000	0.042	1.2
tubulin, beta 4 (TUBB4A)	21361322	36	0.010	30.000	0.009	0.9
tubulin, beta, 2 (TUBB4B)	5174735	232	0.068	235.000	0.074	1.1
tyrosine 3/tryptophan 5 -monooxygenase activation protein, zeta polypeptide (YWHAZ)	4507953	4	0.001	3.000	0.001	0.8
valosin-containing protein (VCP)	6005942	16	0.005	9.000	0.003	0.6
voltage-dependent anion channel 2 (VDAC2)	42476281	11	0.003	11.000	0.003	1.1
voltage-dependent anion channel 3 isoform b	25188179	7	0.002	12.000	0.004	1.9

A cut-off of 2-fold difference in *NSC* ratio was considered as significantly differentially expressed in *ROS* +. Overexpressed and underexpressed proteins are shown in bold.

### Gene ontology annotations of the proteins identified in ROS+ and ROS- samples

**Figure 1:**
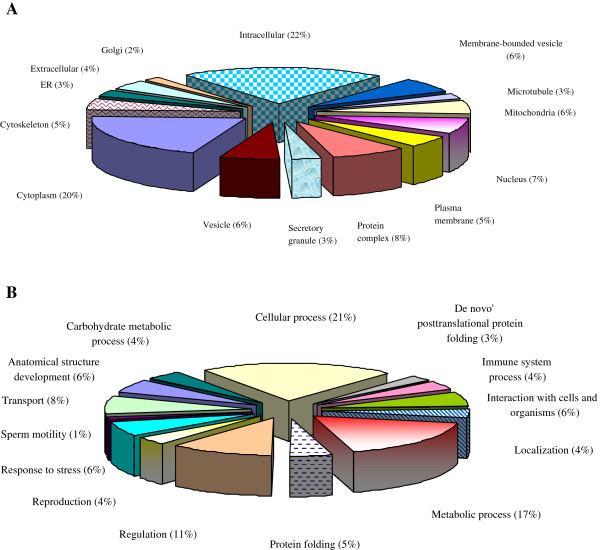
**Functional annotations from consolidated publicly available software tools A: Cellular distribution showing the maximum distribution was intracellular (22%) followed by cytoplasmic distribution; B: Biological processes distribution of proteins identified in spermatozoa from ROS+ and ROS- group showing the highest distribution was in the cellular processes (21%) followed by metabolic processes.**

The cellular distributions of the 74 proteins present in the ROS+ and ROS- samples showed that there was a greater distribution of proteins in the intracellular region compared to the extracellular region (Figure 1A). A significant number of proteins were also distributed in the cytoplasmic region. The proteins involved in the biological processes showed a greater distribution in cellular processes (21%) followed by metabolic processes (17%) (Figure 1B).

### Gene ontology annotation comparisons of the differentially expressed proteins in ROS+ and ROS- samples

**Figure 2:**
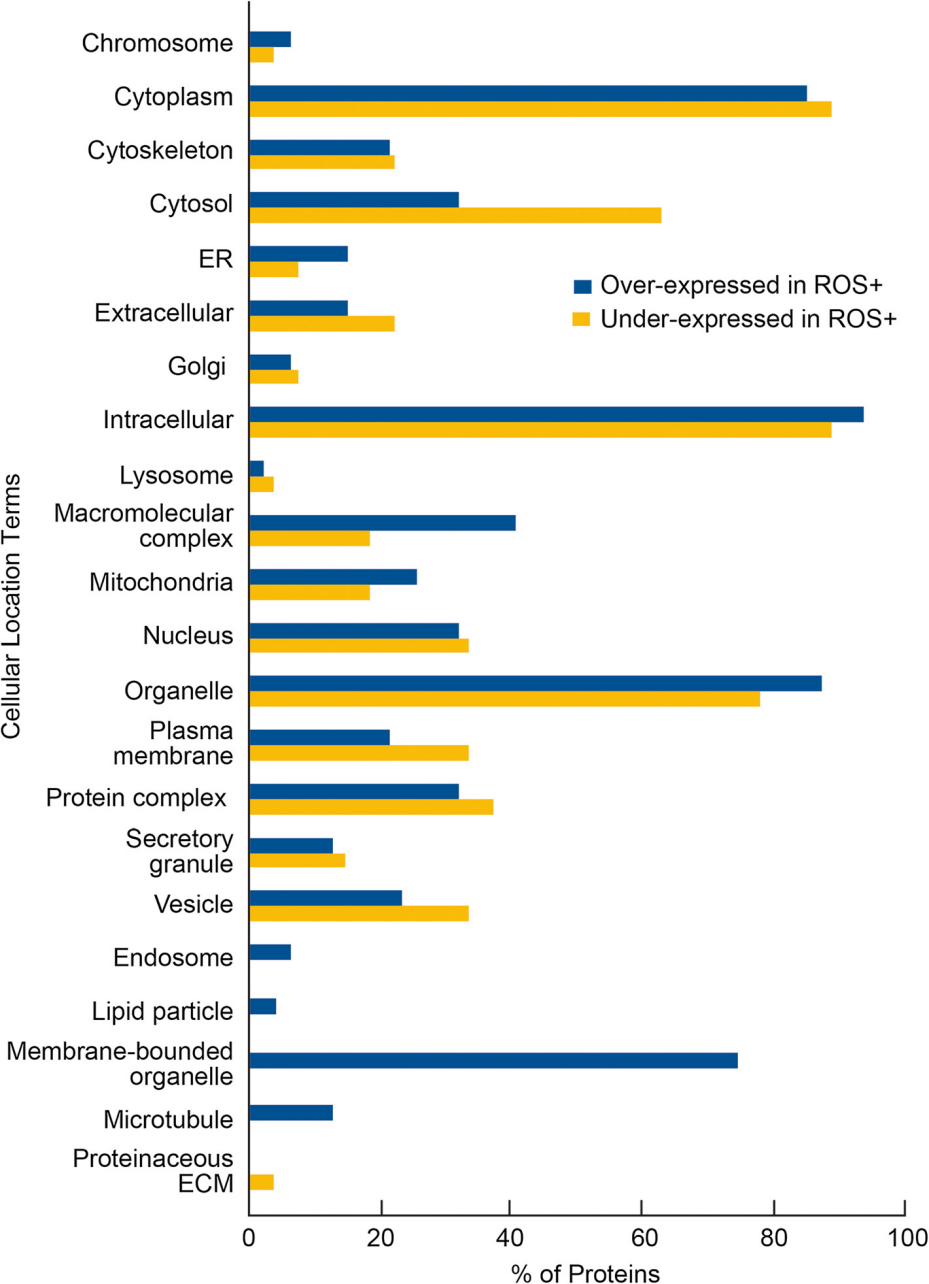
Cellular distribution showing the proteins that were significantly overexpressed in ROS + group were located in the cytoplasm, intracellular, organelle and membrane-bound organelle and underexpressed in ROS + group were located in the cytoplasm, cytosol, intracellular and organellar.

Based on the Gene Ontology classifications, the majority of the differentially expressed proteins that were over or underexpressed in ROS+ group compared to the ROS- group were localized to the cytoplasmic and intracellular regions (Figure 2). Specifically, the cellular compartments of intracellular, organelle, macromolecular complex region and mitochondria were predominantly occupied by the overexpressed proteins. The cytosolic, cytoplasm, extracellular, plasma membrane, protein complex and the vesicular region showed an abundance of underexpressed proteins when compared to the overexpressed ones. Strikingly, the endosome, lipid particle, membrane-bound organelles and the microtubules showed only the presence of overexpressed proteins in the ROS+ group while the proteinaceous extracellular matrix was restricted only to the underexpressed proteins.

**Figure 3:**
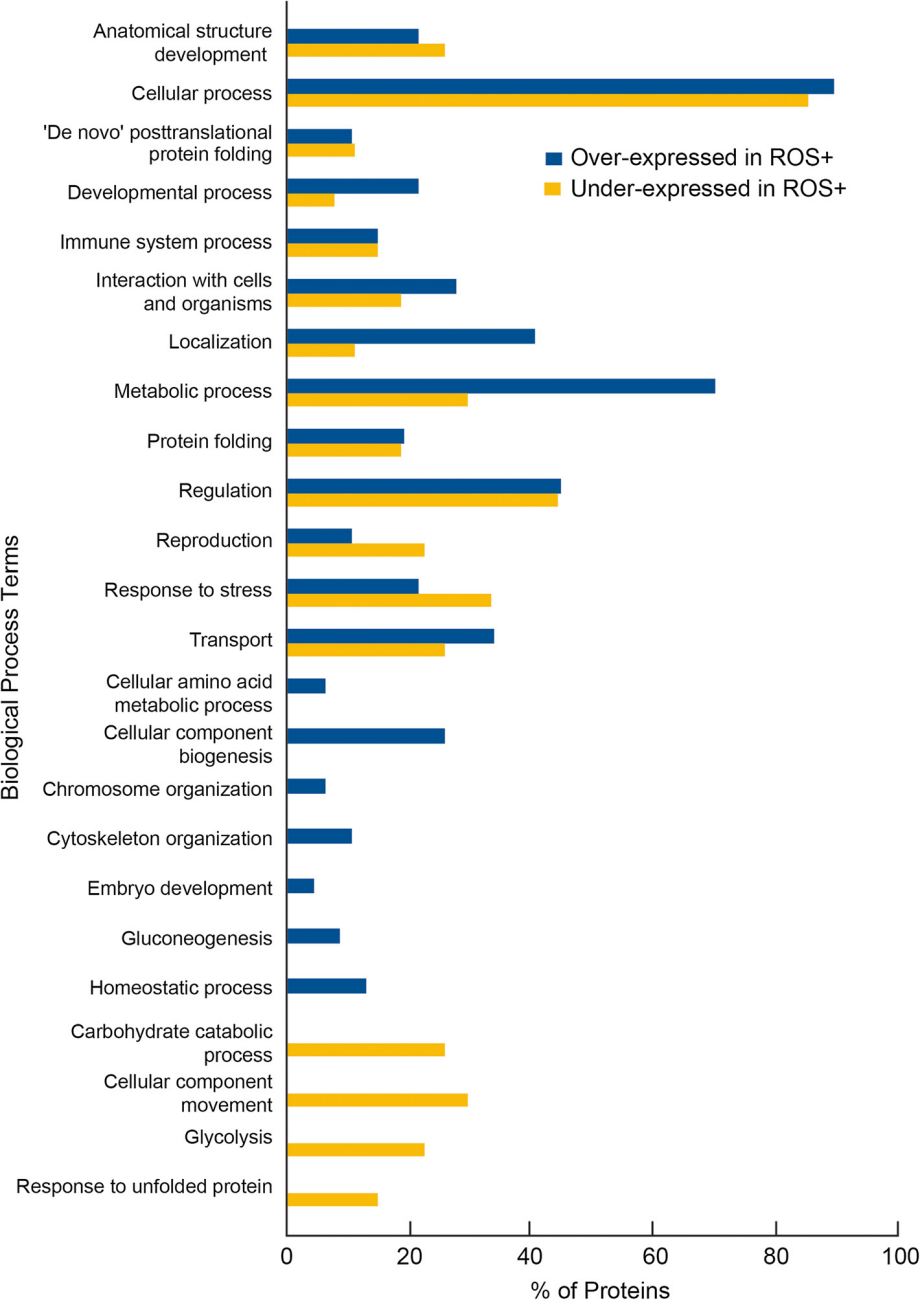
Biological process distribution of proteins of overexpressed were cellular processes, metabolic processes, localization, regulation and transport in spermatozoa from ROS + compared to cellular preocesses, regulation, response to stress, cellular movement and glycolysis in ROS- group.

GO analysis also revealed that the majority of over and under expressed proteins in the ROS+ group was found to be involved in cellular processes (Figure 3). The proteins involved in developmental processes, cellular process, interactions with cells and organisms, localization, metabolic processes and transport were common to both under and overexpressed proteins. Overexpressed proteins were abundantly expressed for these functions. Many of the processes such as cellular amino acid metabolic processes, cellular component biogenesis, chromosome organization, cytoskeleton organization, embryo development, gluconeogenesis and homeostatic processes were found to be restricted only to the overexpressed proteins. Similarly, the processes such as carbohydrate catabolic processes, cellular component movement, glycolysis, and response to unfolded protein were found to be restricted to the underexpressed proteins.

### Pathways and network analysis of differentially expressed in ROS+ proteins using Ingenuity Pathway Analysis (IPA) and Metacore™ software packages

A list of significantly enriched or topmost pathways and/or process networks associated with the over and underexpressed proteins in the ROS+ group is shown in Table 3. Cellular processes were comprised of cell cycle, cell adhesion, cellular morphology, cellular movement and cell death and cell survival. Protein modifications included protein folding and degradation; general metabolic pathways were: carbohydrate metabolism (such as glycolysis and gluconeogenesis), amino acid metabolism, energy metabolism and oxidative stress regulation. With respect to the pathways related to carbohydrate metabolism, enzymes operating in gluconeogenesis and glycolysis including phosphoglycerate kinase 2 (PGK2) and glyceraldehyde phosphate dehydrogenase-S (GAPD-S) were found to be underexpressed in the ROS+ group for both gluconeogenesis and glycolysis while glyceraldehyde-3-phosphate dehydrogenase (GAPDH), fructose-biphosphate aldolase A(ALDOA) and mitochondrial malate dehydrogenase precursor (MDH2) were overexpressed. The overexpressed proteins, especially in the glycolytic pathway, were triosephosphate isomerase 1 (isoform 1(TPI1), glyceraldehyde-3 phosphate dehydrogenase (GAPDH) and fructose-biphosphate aldolase A (ALDOA). Two enzymes involved in the fermentation of pyruvate to lactate – L-lactate dehydrogenase C (LDHC) and L-lactate dehydrogenase A isoform 1(LDHA) were underexpressed. In addition, two enzymes functional in sucrose degradation pathways - triosephosphate isomerase 1 (TPI1) and fructose biphosphate aldolase A (ALDO A), were overexpressed.

**Figure 4:**
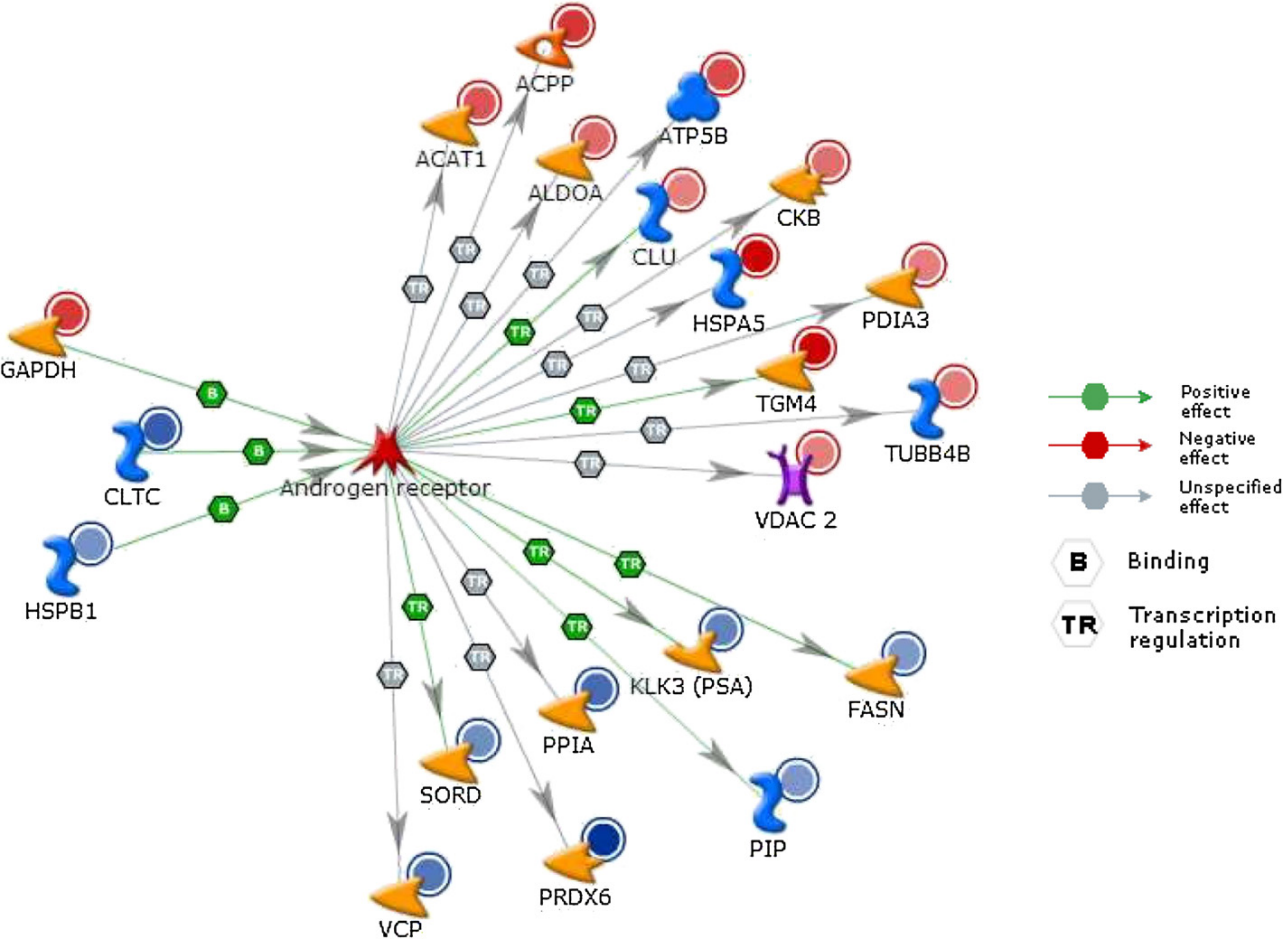
**Transcriptional regulatory network showing interactions between differentially expressed ROS + proteins and androgen receptor.** Proteins with red or blue circles around them are over-expressed (HSPA5 and TGM4) or underexpressed (PRDX6) in spermatozoa from the ROS + (relative to ROS-) group. The levels of expression values are reflected in the intensity of red or blue colors. Green arrows with a hexagon indicate positive effect. TR = Transcription regulation; PGK2 = phosphoglycerate kinase 2; GAPD-S = glyceraldehyde phosphate dehydrogenase-S; GAPDH = glyceraldehyde-3-phosphate dehydrogenase; ALDOA = fructose-biphosphate aldolase A; and MDH2 = mitochondrial malate dehydrogenase precursor.

**Figure 5:**
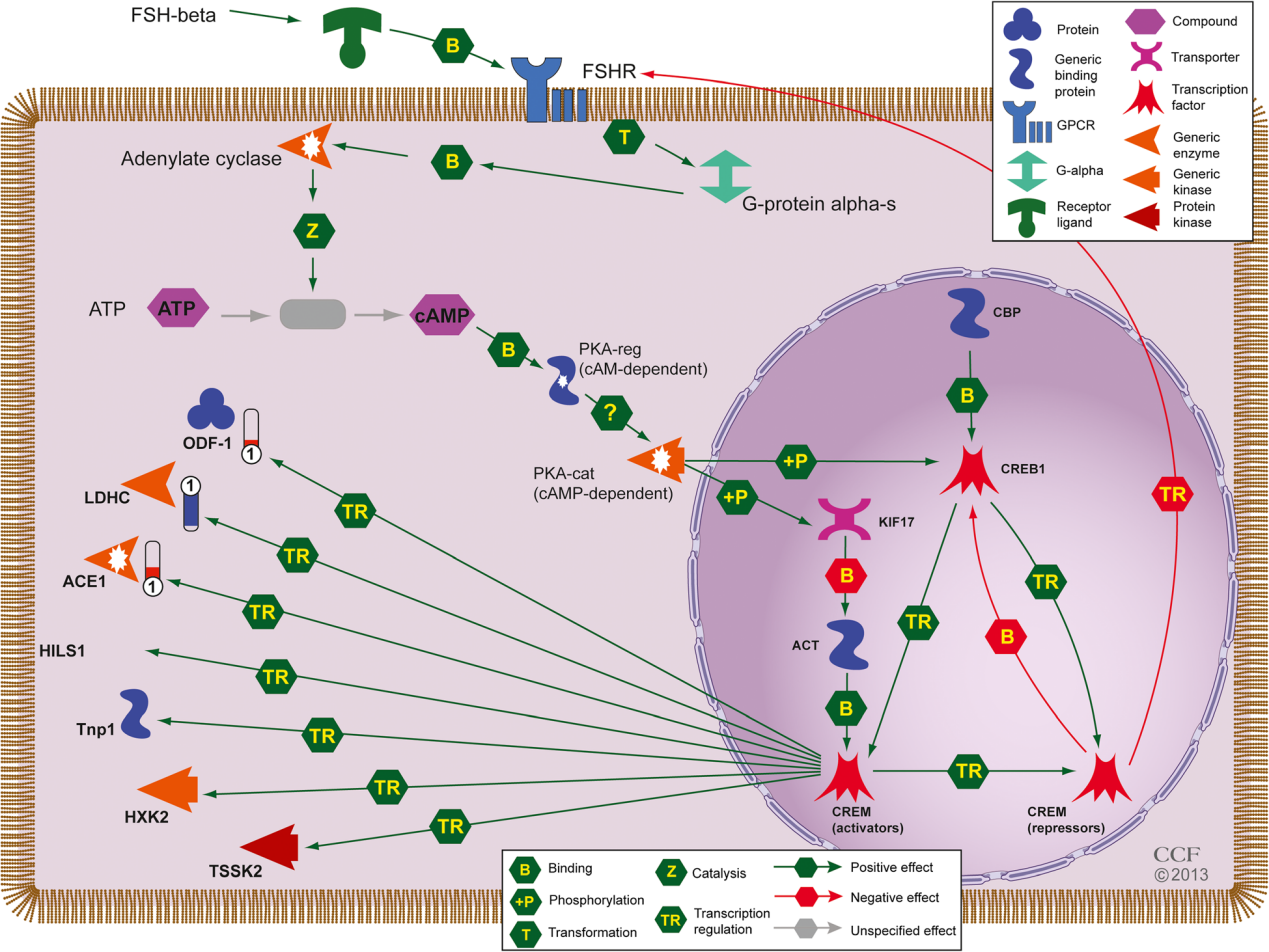
**CREM signaling in the testis.** Proteins in red or blue are the ones over or underexpressed in spermatozoa from the ROS + compared to ROS- group. The color levels in the tubes reflect their expression levels (red-overexpressed and blue underexpressed). B = Binding; Z = catalysis; T = transformation and TR = transcription regulation. The figure shows the generic enxzymes (Adenylate cyclase, LDHC, ACE1, HXK2); Protein kinases (PKA-cat (cAMP –dependent and TSSK2); CREM activators and repressors; generic binding proteins (ACT, CPB and Tnp1 and PKA-reg (cAMP –dependent) and proteins (HILS1 and ODF1) involved in the CREM signaling in the testis. CREM = cAMP responsive element modulator; ACE = Angiotensin I converting isoform 1 precursor; ODF1 = outer dense fiber of sperm tails 1; LDHC = L- lactate dehydrogenase C.

Transcriptional regulatory network analysis of the differentially expressed proteins, using Metacore™, showed that the androgen receptor was one of the topmost regulators with 21 differentially expressed proteins in the ROS+ group interacting with the receptor (Figure 4). As a result of the established connection between germ cell development and the levels of androgens, it was important to elucidate the potential interaction of these over- and underexpressed proteins with the androgen receptor. One of the pathways that may be relevant to spermatogenesis is cAMP responsive element modulator (CREM) signaling in the testis (Figure 5). In this pathway, 3 proteins were found to be differentially expressed. Angiotensin I converting isoform 1 precursor (ACE). The outer dense fiber of sperm tails 1 (ODF1) was found to be underexpressed whereas the L- lactate dehydrogenase C (LDHC) was overexpressed in ROS+ sample compared with ROS- sample.

## Discussion

Oxidative stress plays a key role in the etiology of male infertility, possibly by altering protein expression levels in spermatozoa, causing molecular and genetic defects. Therefore, understanding the function of each protein involved and the post-translational modifications that occur during sperm-maturation is important and may lead to a useful biomarker for male infertility. In earlier studies, we demonstrated that ROS are independent of the semen parameters [6]. Therefore, in the current study, we compared the sperm proteome from a group of men with high levels of ROS (ROS+) with that from men with low or physiological levels of ROS (ROS-). The ROS grouping was done irrespective of the patient’s clinical diagnoses and semen parameters: the only criteria was the presence or absence of ROS.

We have identified 74 proteins, 15 of which had a >2-fold difference in their expression levels in the ROS+ group when compared to the ROS- group. Based on the spectral counts of the 74 proteins, we identified 47 were overexpressed and 27 that were underexpressed in the ROS+ group compared to the ROS- group. Of the 74 proteins, 17 were determined to be either in their precursor or pre-protein form. The presence of these incompletely processed proteins is indicative of post-translational processing problems as reported by some investigators [31,32]. Accumulation of precursor forms of protein may result in deregulation of the downstream functions of the mature protein [31]. Oxidative stress may result in the accumulation of precursors or preproteins. The cellular distribution of all the proteins as ascertained by GO analysis (Figure 1A) suggested that the majority of the proteins (68 proteins) were intracellular or organellar (62 proteins) in origin, whereas a few were localized in the extracellular (13 proteins) region. The presence of extracellular proteins may be attributed to the presence of round cells in the ejaculate from both the donors and patients. We did not remove these from subsequent processing for proteomic analysis.

Similar comparative studies have been performed previously by other groups in an effort to understand the sperm proteome [31,33,34]. Most notable amongst these are two studies where the expression profile of proteins was compared in asthenozoospermic and normozoospermic donors [31,33]. In these studies, 10 proteins and 17 spots were identified using 2-DE suggesting their role in motility-related male infertility. Xu et al conducted a comparative study between infertile patients and normozoospermic group and identified 24 differentially expressed proteins that could potentially serve as diagnostic markers in identifying the underlying pathology of male infertility [35]. Our findings in this study are novel, as we have compared and identified the proteins that are differentially expressed in spermatozoa obtained from seminal ejaculates with high levels of ROS (ROS+) to those normal levels of ROS (ROS-).

On examining the association of the differentially expressed proteins with the biological processes (Figure 1B), we concluded that the majority of the proteins were involved in cellular, metabolic, and regulatory processes. A smaller distribution of proteins (3-5%) was also found in biological processes such as de novo post translational (8 proteins) and protein folding (14 proteins), and these were identified as ‘enriched processes’ for our list of proteins, by the GO Term Finder.

Even though the spermatozoa become transcriptionally inactive after spermatogenesis, we found the presence of the translational proteins and this may be indicative of the presence of some leftovers from inefficient spermatogenesis. They may also have a role in the normal physiology of the spermatozoa or affect fertilization and embryo development. Further validation through the pathway and process network analysis using IPA and Metacore™, suggested that the topmost function of the differentially expressed proteins was carbohydrate metabolism and included pathways of gluconeogenesis, glycolysis, fermentation of pyruvate to lactate and sucrose degradation. There is a great controversy pertaining to the major pathway of energy metabolism during sperm motility [36-39]. In this context, our findings showed that while oxidative phosphorylation is important for sperm function, the major pathway for energy production for sperm motility was via the glycolytic pathway. To support this view, various respiratory enzymes involved in the glycolytic pathway including phosphoglycerate kinase 2 (PGK2), glyceraldehyde-3phosphate dehydrogenase (GAPDH), fructose- biphosphate aldolase A (ALDOA), glyceraldehyde-3 phosphate dehydrogenase-S (GAPDHS), triosephosphate isomerase 1 isoform (TPI1) were identified in our study. Furthermore, GAPDH-S protein was overexpressed in the ROS+ sample. Oxidation of GAPDH-S as a result of H_2_O_2_ generation has been reported to decrease sperm motility and inhibit GAPDH-S activity [40].

Various isoforms of histone proteins were also identified and these included histone cluster 1, H2aa (HIST1H2AA), histone cluster1 H2ae (HISTH1AE) and histone cluster1 and H2ba (HIST1H2BA). Histones are a group of proteins that are replaced by the protamines during sperm maturation in the epididymal region [41]. Their presence in the ejaculated spermatozoa is indicative of improper packaging of sperm chromatin and subsequent DNA damage. These findings have been attributed to oxidative stress [5,41].

We found 17 precursor proteins to be dysregulated; of these, 10 were over-expressed and 5 were underexpressed with a 2-fold difference between the ROS+ and ROS- groups. The differentially expressed proteins were mainly intracellular, cytoplasmic or organellar in distribution. This is contrary to the findings of Xu et al, who reported a higher distribution of differentially expressed proteins in the extracellular region in the infertile groups with normal semen parameters [35]. This difference may be attributed to the import/export of epididymal and seminal vesicle proteins. Furthermore, the distribution of overexpressed and underexpressed proteins in the ROS+ group revealed their involvement in various processes. We noted that many proteins showing significant changes in expression between the ROS+ and ROS- groups had roles in sexual reproduction, metabolic processes and stress response (Figures 2 and 3).

Our data suggests that semenogelin II precursor (SEMG2), which is involved in sexual reproduction, was underexpressed in the ROS+ group. Overexpression of semenogelin I and II precursor has been reported in previous comparative studies [31,35]. Xu et al reported an increase in both isoforms of semenogelin in the spermatozoa of infertile patients compared to the normozoospermic men [35]. Semenogelin -1 and semenogelin -2 have been reported in men with varicocele only as well as in men with varicocele with a history of moderate as well as heavy smoking. Semenogelin is known to cause a decrease in sperm motility, but it was within the normal range in the ROS+ samples. Semenogelin (SEMG) is the major protein of seminal fluid proteins (20-40%) and is secreted from seminal vesicles, epididymis, and prostate [42,43]. It is an androgen dependent protein that exists in two forms I (52 kDa) and II (71-76 kDa). SEMG helps in the formation of the semen coagulum that is degraded later by prostate-specific antigen. SEMG counteracts oxidative stress by reducing the generation of free radicals through several mechanisms: slow sperm motility of the entrapped sperm, reduced energy consumption, free radical generation and inhibition of superoxide radical generation through direct interference with sperm NADH oxidase [44].

A great number of proteins are involved in the energy production required for the spermatozoon tail movement. We identified several proteins that are involved in energy producing processes such as the carbohydrate metabolic pathways of glycolysis and gluconeogenesis. Enolase I (ENO1) was found to be significantly underexpressed while respiratory enzyme mitochondrial malate dehydrogenase precursor (MDH2) was found to be overexpressed. MDH2 is an ROS producing enzyme localized in the sperm mitochondria [45]. It catalyzes the last step of the Krebs cycle when malate is converted into oxaloacetate using NAD+/NADH as intermediates. Several of the chaperone proteins such as heat shock protein 90 kDa beta, member 1 (HSP90B1) and heat shock 70 kDa protein 5 (HSPA5) were also seen to be overexpressed in response to stress. The association of these proteins with various processes related to cellular stressors such as heat, glucose deprivation, free radical attack, and infection indicates that ROS induces a stress response in the spermatozoa [46-48].

The GO annotations further revealed the involvement of overexpressed proteins in cellular amino acid metabolic processes. Examples of proteins that were found to be overexpressed included transglutaminases (TGM4) and glutamine synthetases. Transglutaminases are a family of calcium-dependent enzymes that catalyze the post-translational modification of the selected glutamine residues on proteins by cross-linking with the peptide-bound lysine residues or incorporating polyamines [49,50]. Transglutaminase that is secreted from the prostate is known as TGM4 prostate. A recent study showed increased levels of transglutaminase in response to oxidative stress in the prostate [51]. Excessive production of ROS has been reported to induce cell senescence, oxidative protein modifications, and cell membrane damage, increase intracellular calcium and activate certain cytokines such as TGFb [51]. Oxidative stress impacts the aggregation, increased molecular weight, unfolding, denaturation and proteolysis of cellular proteins. These effects may result in the initiation of cellular hyperamonemia, which probably triggers the expression of glutamate synthetase to redirect the production of the released ammonium into glutamate [52].

Glutathione peroxidase 4 isoform A precursor (GPX4) and peroxiredoxin 6 (PRDX6) function to reduce oxidative stress. The former was overexpressed and the latter was underexpressed in the ROS+ group. GPX4 is a well-known antioxidant enzyme and its increased expression in sperm in the ROS+ samples is reflective of its influence on genes synthesizing this enzyme. PRDX6 also belongs to a family of antioxidant enzyme known as peroxiredoxins. They detoxify H_2_O_2_ similar to catalase or glutathione peroxidases (GPXs) [53,54]. Peroxiredoxin 6 is localized in the sperm head (postacrosomal region and equatorial segment) and sperm tail [55]. Reduced levels of peroxiredoxins (PRDX1 and PRDX6) have been reported in infertile men with oxidative stress. Reduced levels of PRDX6 have also been reported in the seminal plasma from men with varicocele. Differential amounts of peroxiredoxin 6 have been reported (41% in men with varicocele and 65% in men with idiopathic infertility) [56]. Human spermatozoa are reported to have high sensitivity to ROS and may be a result of reduced recycling of peroxiredoxins after exposure to oxidative stress [55].

Optimal levels of expression and function of androgens are central to the process of spermatogenesis. To understand the dependency of the differentially expressed proteins in the ROS+ groups, a network was derived to identify proteins regulated by androgen receptors. Spermatogenesis is dependent on androgen action and androgens act by stimulating the receptors present on the Sertoli cells [57,58]. Transcriptional network analysis (Figure 4) showed that 21 proteins were regulated by the androgen receptor. In our study, several proteins appeared to be transcriptionally activated by the androgen receptor but the effect of the androgen receptor on several other proteins is still unknown (Figure 5). Stanton et al reported that any loss to the androgen stimulus during meiosis induces changes in the proteins that are associated with various molecular processes such as apoptosis, cell signaling, oxidative stress and RNA processing [58]. These investigators further validated this observation by immunostaining for oxidized DNA adducts – they showed that spermatocytes that undergo oxidative stress induced DNA damage during androgen suppression. The completion of meiosis requires androgen action via the Sertoli cell [59]. Low levels of testicular androgens support meiosis while a high level is required for spermiogenesis.

Testicular testosterone suppression has been shown to adversely influence antioxidant activity [60]. The present study utilizing Network analysis revealed heat shock protein beta-1 (HSPB1) and clathrin heavy chain 1 (CLTC), which are known to bind and activate androgen receptor were actually underexpressed in the ROS+ group. We also observed that GAPDH levels were overexpressed, and that it was bound with the androgen receptor to impart a stimulatory effect in the ROS+ sample. The mechanism by which the androgen stimulus is transduced by the Sertoli cell to the germ cells is still unclear. In addition, it is well known that in majority of the infertility cases attributed to a male factor, spermatogenic arrest is a common feature. The process of spermatogenesis is dependent on the cAMP responsive element modulator (CREM) signaling pathway in the testis which operates in coordination with the CREM modulators (repressors and activators) [61]. CREM is essential for male fertility and the absence of CREM-dependent transcription in post-meiotic germ cells is known to result in an arrest of spermatid differentiation and apoptosis [62,63]. CREM repressors are expressed during pre-meiotic germ cells while CREM activators are expressed in the post-meiotic germ cells. In order to understand the impact of oxidative stress on impaired spermatogenesis, we examined the CREM signaling in testis. Our findings revealed that three of the identified proteins that were differentially expressed in the ROS+ group were modulated by CREM activators. This included ODF1 and ACE1 proteins that were underexpressed as well LDHC, which was overexpressed. These findings suggest that oxidative stress may affect the switching of genes at the spermatogenic level. Comparative studies on CREM signaling in testis have been conducted on normo- and oligozoospermic men and men with round spermatids vs. round spermatid maturation arrest [64]. These findings further support the fact that a lack of a switch in the expression of CREM gene isoforms may have an adverse impact on spermatogenesis in humans.

To summarize, in this study, we have identified various proteins that are implicated in a multitude of functions associated with response and management of oxidative stress. The increased expression of histone cluster 1H2ba (HIST1H2BA), mitochondrial malate dehydrogenase precursor (MDH2), transglutaminase 4 (TGM4), glutathione peroxidase 4 isoform A precursor (GPX4), glutamine synthetase (GLUL), heat shock proteins (HSP90B1 and HSPA5) in the ROS+ sample suggests that these proteins can serve as potential biomarkers of oxidative stress. Thus alterations reported in our study in men with and without oxidative stress may help explain pathways leading to the altered semen phenotype especially in men exhibiting oxidative stress both in infertile men as well as in infertile men with varicocele. However, the interaction between the proteins and the androgen receptors is still unclear and warrants further investigation. It is important to accurately assess the protein levels and verify that the differentially expressed proteins are indeed involved in the process of oxidative stress and are possibly modified as a result of oxidative stress. In future studies, as a follow-up to the present study, quantitative estimation of the differentially (overexpressed and underexpressed) expressed proteins, utilizing western blotting and antibodies is warranted. The findings of our study provide the groundwork for further testing including the proposition that these newly identified sperm proteins play crucial roles in oxidative stress and the pathophysiology of male infertility.

## Competing interests

The authors declare that they have no competing interests.

## Authors’ contributions

RK: participated in the study conception/design, review of the data and writing of the manuscript and final approval; SD: acquisition and preparation of samples for analysis; BW, acquisition of samples for analysis; interpretation of the results; SY: acquisition of the samples, interpretation of the data, discussion o results; BG: contributed to bioinformatic analysis, data interpretation and participated in the paper redaction; AH: drafting of the article; GM participated in the review of the data and writing of the manuscript; AA contributed to the study design, and review of the data. All authors read and approved the final manuscript.
